# Minimal handgrip force is needed for transcutaneous electrical stimulation to improve hand functions of patients with severe spinal cord injury

**DOI:** 10.1038/s41598-022-11306-5

**Published:** 2022-05-11

**Authors:** Ruyi Huang, Ali A. Nikooyan, Lisa D. Moore, Sharon Zdunowski, Erika Morikawa, Tiffany Sierro, Dimitry Sayenko, Parag Gad, Tali Homsey, Timothy Le, Meghna A. Madhavan, Marina Abdelshahid, Martina Abdelshahid, Yan Zhou, Mark R. Nuwer, Majid Sarrafzadeh, V. Reggie Edgerton, James C. Leiter, Daniel C. Lu

**Affiliations:** 1 Neurosurgery Department, David Geffen School of Medicine, University of California, Los Angeles, Los Angeles, CA 90095 USA; 2 Neuroplasticity and Repair Laboratory, University of California, Los Angeles, Los Angeles, CA 90095 USA; 3 Brain Research Institute, University of California, Los Angeles, Los Angeles, CA 90095 USA; 4 Department of Integrative Biology and Physiology, University of California, Los Angeles, Los Angeles, CA 90095 USA; 5 Department of Computer Science, University of California, Los Angeles, Los Angeles, CA 90095 USA; 6 Department of Neurobiology, University of California, Los Angeles, Los Angeles, CA 90095 USA; 7 Department of Orthopedic Surgery, University of California, Los Angeles, Los Angeles, CA 90095 USA; 8 Neuromotor Recovery and Rehabilitation Center, University of California, Los Angeles, Los Angeles, CA 90095 USA; 9 White River Junction VA Medical Center, White River Junction, VT 05009 USA; 10 School of Information, University of California, Berkeley, Berkeley, CA 94720 USA; 11 Houston Methodist Hospital, Houston, TX 77030 USA

**Keywords:** Neuroscience, Motor control, Spinal cord

## Abstract

Spinal cord stimulation enhanced restoration of motor function following spinal cord injury (SCI) in unblinded studies. To determine whether training combined with transcutaneous electrical spinal cord stimulation (tSCS), with or without systemic serotonergic treatment with buspirone (busp), could improve hand function in individuals with severe hand paralysis following SCI, we assessed ten subjects in a double-blind, sham-controlled, crossover study. All treatments—busp, tSCS, and the busp plus tSCS—reduced muscle tone and spasm frequency. Buspirone did not have any discernible impact on grip force or manual dexterity when administered alone or in combination with tSCS. In contrast, grip force, sinusoidal force generation and grip-release rate improved significantly after 6 weeks of tSCS in 5 out of 10 subjects who had residual grip force within the range of 0.1–1.5 N at the baseline evaluation. Improved hand function was sustained in subjects with residual grip force 2–5 months after the tSCS and buspirone treatment. We conclude that tSCS combined with training improves hand strength and manual dexterity in subjects with SCI who have residual grip strength greater than 0.1 N. Buspirone did not significantly improve the hand function nor add to the effect of stimulation.

## Introduction

Cervical spinal cord injury (SCI) is associated with a drastic reduction of upper extremity function, especially in the hands^1–3^. Decreased hand strength and dexterity after cervical SCI significantly impairs quality of life and the capacity for independent living^4–9^. Patients with motor-complete injuries often maintain some functional supraspinal connections caudal to the injury. Although the brain may still be able to communicate with paralyzed muscles, these spared supraspinal connections are insufficient to activate paralyzed limbs in the absence of additional therapy. Non-invasive electrical stimulation on the cervical spine using transcutaneous electrodes or transcutaneous spinal cord stimulation (tSCS) has improved upper extremity strength and control. Prior investigations of transcutaneous electrical spinal cord stimulation mainly evaluated the maximum gripping force, but other voluntary hand functions such as dexterity were not evaluated. Furthermore, in previous studies on subjects with motor-complete and motor-incomplete injuries, the sustained effects on upper extremity were not evaluated post-treatments^10,11^. Several studies suggest that the co-administration of serotonergic agonists can enhance the effects of spinal cord stimulation and improve functional recovery^12,13^. Buspirone, a direct serotonin 1A (5-HT_1A_) receptor agonist with minimal side effects, augmented the effects of spinal cord stimulation in tetraplegic subjects who recovered control of the hands^10^. Furthermore, buspirone facilitated the neural plasticity in motor recovery after the spinal cord injuries^14^. Therefore, in this study, we investigated the effects of tSCS and buspirone on both gripping force and hand dexterity as well as the lasting effect after the completion of treatments. We compared the separate and combined effects of tSCS and buspirone on voluntary hand function in an evaluator blinded, sham-controlled, crossover study in subjects with severe, motor complete cervical SCIs (Table 1).

**Table 1:** Demographic and baseline AIS score as well as the mean, SD and N of maximum grip force (in Newtons) across all baseline trials (6 weeks) for each subject.

ID	Group	Sex	Age	Level	Years post injury	AIS	Maximum grip force mean (N)	SD ( ±)	Number of baseline trials
S1	NF	M	62	C3	4	A	0.0037	0.0032	36
S2	NF	F	22	C4	4	B	0.0095	0.0075	54
S3	NF	M	63	C5	4	A	0.0075	0.0016	54
S4	NF	M	27	C6	4	A	0.0215	0.0071	33
S5	NF	F	24	C5	8	B	0.0126	0.0039	72
S6	F	F	22	C7	9	A	0.2038	0.0707	72
S7	F	F	26	C4	4	B	0.3450	0.0721	54
S8	F	M	28	C5	4	B	1.0929	0.2235	36
S9	F	M	40	C6	14	B	1.2636	0.2618	81
S10	F	M	30	C5	2	A	1.3434	0.2873	36

## Materials and methods

This study was registered on ClinicalTrials.gov (NCT02313194, first registration: 09/12/2014) and approved by the Institutional Review Board at the University of California, Los Angeles (IRB 12-001416) and the Food and Drug Administration (IDE G140103; IND 132651). During the testing phase, each subject underwent 3 months of open-label buspirone dose escalation (5/10/15 mg oral, twice a day, Table 2). All subjects gave written, informed consent prior to enrollment and met all inclusion criteria and none of the exclusion criteria (Table 3)^10^. All experiments were performed in accordance with the Declaration of Helsinki and were compliant with HIPAA.

**Table 2:** Formulation of Buspirone and Placebo.

	Buspirone	Placebo
Active Ingredient	BUSPIRONE HYDROCHLORIDE (UNII: 20 7LT9 J9OC) (BUSPIRONE -UNII:TK6 5WKS8HL)	N/A
Other ingredient	ANHYDROUS LACTOSE (UNII: 3SY5LH9 PMK)	To be determined by UCLA Investigational Pharmacy
	SILICON DIOXIDE (UNII: ETJ7Z6XBU4)	
	MAGNESIUM STEARATE (UNII: 70 0 9 7M6 I30 )	
	CELLULOSE, MICROCRYSTALLINE (UNII: OP1R32D6 1U)	
	SODIUM STARCH GLYCOLATE TYPE A POTATO (UNII: 58 56 J3G2A2)

**Table 3:** All research participants, irrespective of age or sex, will meet the following criteria^10^.

Inclusion criteria	Exclusion criteria
18 years of age or older	Cardiopulmonary disease or dysautonomia that would contraindicate hand/arm movement
Non-progressive SCI at or above C5 functional level	Recipients of Botox injections in the prior 6 months
Be at least 1-year post injury	Disorders or conditions that would require MRI monitoring
Be unable to grip or move independently, requiring full assistance with all rehabilitation activities and activities of daily living	Coagulopathy, cardiac risk factors, or other significant medical risk factors for surgery
Segmental reflexes remain functional below the lesion (screening for preservation of lower motoneuron innervation)	Prior implantations of neurostimulators, cardiac pacemakers, defibrillators, shunts, stents, or aneurysm clips
Female subjects of child-bearing potential must not be pregnant and must be using a medically acceptable method of contraception	Involved in another clinical trial
	Ongoing treatments with an anti-spasticity medication regimen
	Clinically significant depression or ongoing drug abuse
	Painful musculoskeletal dysfunction, unhealed fracture, contracture, pressure sore, or infection that might interfere with upper extremity training

### Electromyographic activity

Surface electromyographic (EMG) signals were recorded bilaterally from electrodes placed on eight muscles: deltoid (DEL), biceps brachii (BIC), triceps brachii (TRIC), brachioradialis (BRAD), extensor digitorum (ED), flexor digitorum superficialis (FD), hypothenar (HThen), and thenar muscles (Then) with a fixed 17 mm interelectrode distance (Fig. 2a). Ground electrodes were placed bilaterally on the acromion. EMG signals were amplified (Konigsberg Instruments, Pasadena, CA), band pass filtered 10 Hz to 5 kHz and acquired at 10 kHz with a 16 channel A/D board and analyzed with customized LabVIEW software (National Instruments, Austin, TX).

### Hand dynamometer

Each subject’s hand strength and manual dexterity were measured using a hand dynamometer (Handgrip, MediSens Wireless, Santa Clara, CA). The device measured grip force by displaying visual targets and a tracker to represent forces exerted upon the device’s handle. Subjects were asked to squeeze the device in a stereotypical pattern to manipulate a visual tracker during the maximum voluntary contraction (MVC), sine force generation and oscillation between grip and release (OSC) without engaging the arm or wrist for movement assistance. Each subject performed the MVC task by squeezing and holding the dynamometer handle three times for 5 s followed by a 10 s rest within a 45 s period (Fig. 3d). Sine force generation tested grip control and entailed alternatively squeezing and releasing the handle for 45 s while attempting to trace a sinusoidal pattern displayed on the computer screen. We quantified the average distance between the displayed MVC sinusoidal pattern and each subject’s trace to infer fine motor control (Fig. 4a). In the OSC task, the subject squeezed the handle to draw traces on a moving background as rapidly as possible for 10 s while attempting to exceed 75% of the subject’s MVC. The distance the target traveled on the screen was multiplied by the average force the patient generated. The scores for the Sine and OSC tasks were normalized and presented on a scale from 0 to 1 using the following formula:

$${\text{Scores for Sine or OSC }} = \frac{Current trail - minimum trial}{{Max trial - minimum trial}}$$.

### Study design

**Figure 1:**
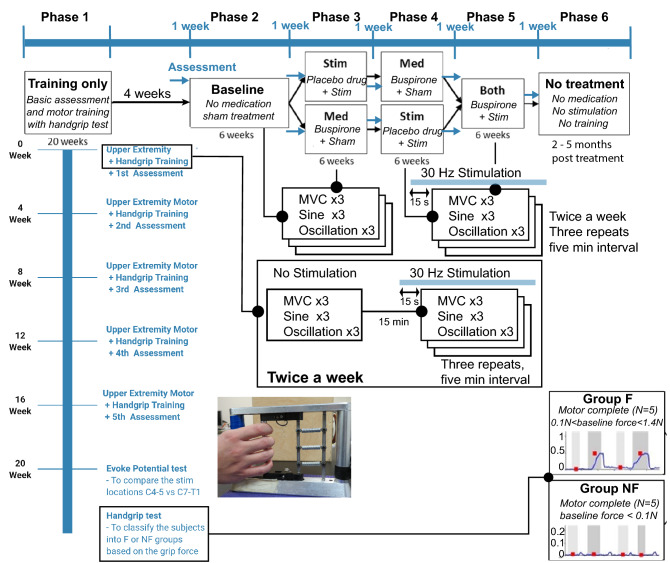
Overview of study design and tasks performed. A single population of subjects was recruited from a prior study that assessed the effect of handgrip training alone^10,26^. Within the recruited population, two groups were defined: one group of subjects had no hand strength (Group NF; < 0.1 N grip force) and the other population had minimal (> 0.1 N and < 1.4 N) grip force (Group F). The study was broken into six phases as shown above. At the time of enrollment, all subjects underwent baseline physiological assessments to map evoked responses from the spinal cord. The blue arrow indicates the 1-week functional assessment between the four treatment phases as well as before the Baseline (Sham only) and after the Both (buspirone + Stim) phase.

The study comprised six phases (Fig. 1): a 20 weeks phase 1 of individualized physical therapy for up to three times per week to establish a baseline assessment. The next four phases of the study were: a placebo + sham tSCS (phase 2), buspirone or tSCS (phase 3), whichever treatment, buspirone or tSCS, that had not previously been used (phase 4), and tSCS + buspirone (phase 5). The order of phases 3 and 4 was randomized. Sham stimulation consisted of perceptible stimulation administered at one-tenth of the treatment stimulation intensity. Subjects took either buspirone (10 mg) twice a day or an identical placebo dispensed by the UCLA Clinical Trial Pharmacy during each study period. The last phase (phase 6) consisted of a 5-month washout period in which no treatment was given to the subjects.

### Study recruitment

**Figure 2:**
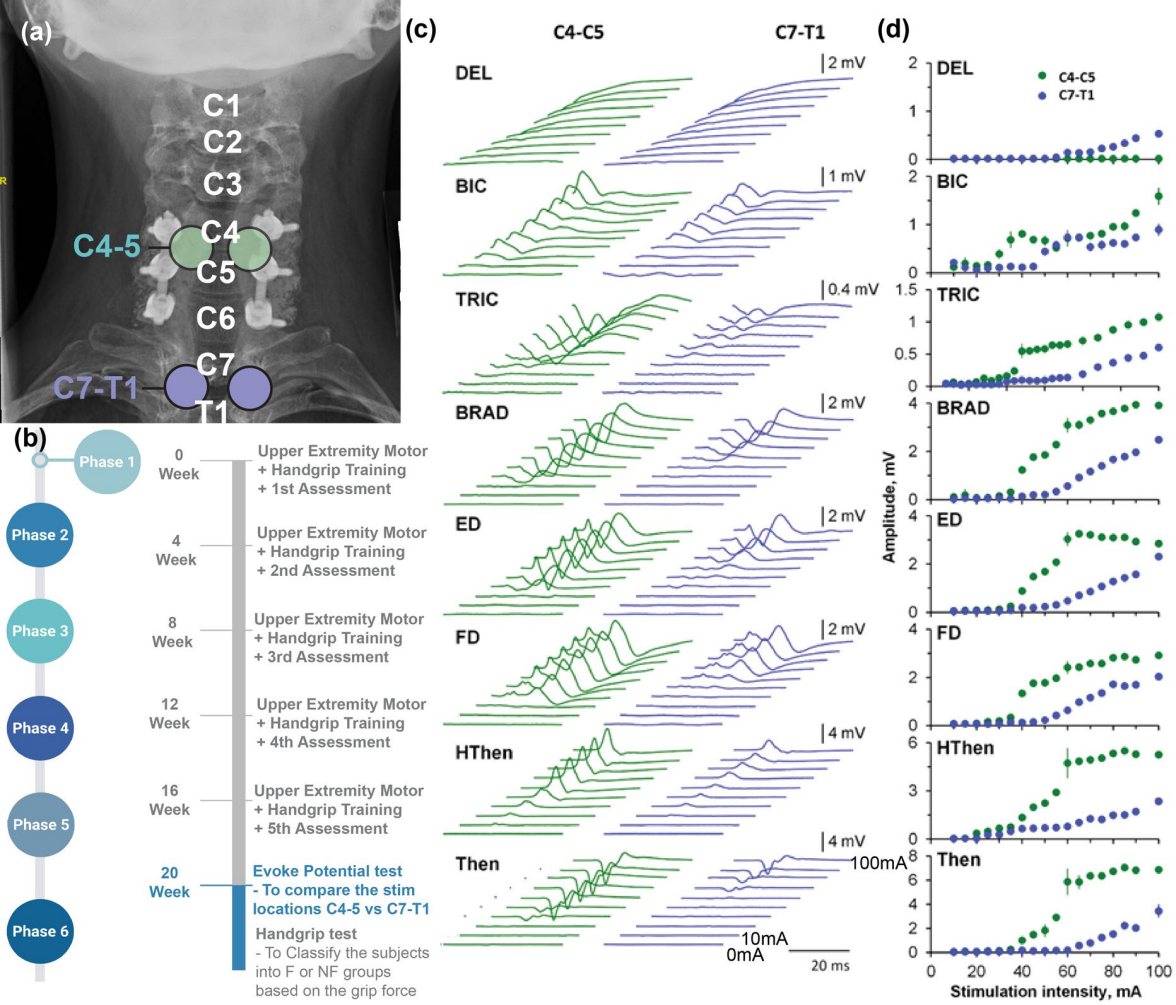
The procedure of determining the stimulation location was based on evoked potential testing. The evoked potential stimulation with 1 Hz was performed after all the hand function training to select the optimal stimulation intensity for each individual subject. (**a**) Two candidate stimulation locations, C4-C5 (Cyan) and C7-T1 (Blue), were tested. (**b**) The evoked potential test was performed during the last week of the 20-week Phase 1 period. (**c**) Representative evoked responses from one participant (S2, AISA A, C4) during tSCS at C4 and C7. The time windows between 5 and 45 ms following the stimulus are shown. (**d**) The recruitment curves of the right arm muscles of S2 at each location of spinal stimulation are shown. DEL, deltoid; BIC, biceps brachii, TRIC, triceps brachii; BRAD, brachioradialis; ED, extensor digitorum; FD, flexor digitorum; HThen, hypothenar; Then, thenar muscles. (**a**) is licensed under Shutterstock (Stock photography company).

We recruited 12 subjects with severe, motor complete, cervical spinal cord injury who received 20 weeks of hand grip physical therapy alone to ensure that the maximal benefit of physical therapy had been obtained, and in whom further improvement during the study could not be attributed to physical training. The number of training sessions varied between 12 and 18 sessions, which was dictated by the unavoidable intercurrent illnesses in the patients. Two subjects were unable to complete the study based on decisions unrelated to the study. Among the remaining 10 subjects, at the end of the Baseline phase after 20 weeks of physical therapy and training of the hand grip test procedures, five subjects generated a grip force between 0.1 and 1.5 N (Group F, Table 1), whereas the remaining 5 subjects could not generate a grip force ≥ 0.1 N (Group NF, Table 1). In all 10 subjects, volitional activation of the flexor digitorum, extensor digitorum, and brachioradialis muscles was confirmed by EMG recordings (Fig. 2).

### Pre-treatment assessment

During Phase 1, subjects performed the MVC, sine, and oscillation tasks during two sessions per week to train the subjects to the tasks. Furthermore, the evoked potential stimulation with 1 Hz were performed after all the hand function training to select the optimal stimulation intensity for each individual subject. These assessment tasks were performed three times consecutively, and the entire series of tasks was repeated three times. Each task was conducted first in the absence of stimulation, followed by three repetitions with stimulation applied at 30 Hz starting 15 s prior to task initiation and maintained at a constant frequency and intensity until the task was completed (Fig. 1). Initially, the tSCS was set at an intensity at which the subject’s hand contracted and generated 25% of the maximum MVC of the patient. In subjects in whom tSCS elicited no measurable displacement of the hand, the maximum tolerable intensity, as well as two lesser intensities (− 20 and − 10 mA) were tested. The stimulation intensity that most effectively increased hand strength and manual dexterity in each subject was selected for the application of genuine stimulation during the study. If a subject requested a reduced stimulation intensity during a study session, stimulation was reduced in increments of 10 percent until the subject was comfortable. A 1-month washout period was observed following these preliminary assessments in Phase 1.

### Treatment with tSCS

Transcutaneous SCS was delivered through round (2.5 cm-diameter), cutaneous, self-adhesive electrodes (ValuTrode Cloth CF3200, Axelgaard Manufacturing Company Ltd., Fallbrook, CA) on the dorsal aspect of the neck at the C4–C5 intervertebral level because this site of stimulation consistently elicited greater bilateral EMG evoked potentials in the eight arm muscles examined than stimulation at C7-T1 (Fig. 2). Stimulation consisted of a 1 ms monophasic rectangular 30 Hz pulses riding on a carrier frequency of 10 kHz generated by a prototype constant current stimulator (NeuroRecovery Technologies 9 Channel External Stimulator, GTX Medical B.V., Eindhoven, The Netherlands)^15,16^. A rectangular (2.5 × 5 cm), cutaneous, self-adhesive electrode was placed over each anterior superior iliac crest, which served as anodes for bilateral stimulation (Fig. 2a). Sham tests were performed with the same set of surface adhesive electrodes for anode and cathode.

### Masking

Study staff who delivered tSCS were blinded to the study details and were not involved in functional assessments or motor training. Research staff who assisted in administering the motor training and the subjects were blinded to drug treatment conditions throughout the study. Because stimulation intensity was noticeably different between sham and treatment stimulation, blinding to stimulation condition was not possible. Staff read instructions from a standardized script to avoid influencing the subject.

### Functional assessments

In addition to the motor tasks, we performed standardized measures of motor-sensory function, spasticity, and evoked potential mapping to set stimulation parameters for later testing. We assessed the overall disability, motor lesion grade, precise hand function, and spasticity of each subject at baseline and following each treatment phase using standardized tests. To assess overall disability, we used the American Spinal Cord Injury Association (ASIA) Impairment Scale (AIS, Table 1) and the International Standards for Neurological Classification of Spinal Cord Injury (ISNCSCI measurement; Fig. 7). Precise assessment of hand function was performed with the Graded Redefined Assessment of Strength, Sensibility, and Prehension (GRASSP 1.0)^17,18^. The Spinal Cord Independence Measure (SCIM)^19^ and the Capabilities of Upper Extremity Questionnaire (CUE-Q) were used to confirm each patient’s level of function^2^. Spasticity of all eight muscles was assessed using the Modified Ashworth Scale and the Penn Spasm Severity/ Frequency Scale^20–22^. We also adapted a Visual Analog Scale (VAS) to assess spasticity: 0 cm corresponded to no spasticity in the prior 24 h, whereas 10 cm corresponded to the worst possible spasticity. The distance measured was converted to a percentage of the total line length to account for discrepancies in total line lengths related to different magnifications generated by different printers^23^. Subjects completed the VAS assessment each day for 7 days prior to each of the four treatment phases as well as 7 days after the end of the last treatment phase (Both).

### Statistical analysis

Outcome measures from this study included handgrip strength (MVC), fine control of the handgrip force (Sine), grip and relax agility (OSC), EMG amplitude and functional motor tests. For sample size and power considerations, our preliminary data on grip strength in n = 5 subjects showed a three-fold mean improvement with a standard error of 20%. Based on this, a sample of n = 5 provides over 80% power using the usual *P* < 0.05 two-sided significance criterion. Data collected during the last three motor training sessions for each subject in each treatment phase 2–5 were analyzed. Shapiro–Wilk testing was performed to check the normality of the data distributions. For handgrip measurements, a three-way analysis of variance (ANOVA) with repeated measures was used with group (F/NF) as a between-subjects factor and phase and sessions as within-subject factors. For the functional tests, a two-way ANOVA with repeated measures was used with group and phase as within- and between-subject factors, respectively. We used Tukey’s test for pairwise comparisons to keep the experiment-wise Type I error < 0.05. The significance threshold was *P* = 0.05. Statistical analysis was performed in Python 3.7. All data are reported as mean ± standard error of the mean.

## Results

The demographic features, level of SCI, severity of injury and maximum baseline grip force are shown in Table 1. Two groups of patients were defined based on the grip force: those with a grip force < 0.1 N and those with a grip force between 0.1 and 1.5 N; the difference in average grip force was significant (*P* = 0.008). None of the other features shown in Table 1 differed between the two groups.

### Primary outcomes

**Figure 3:**
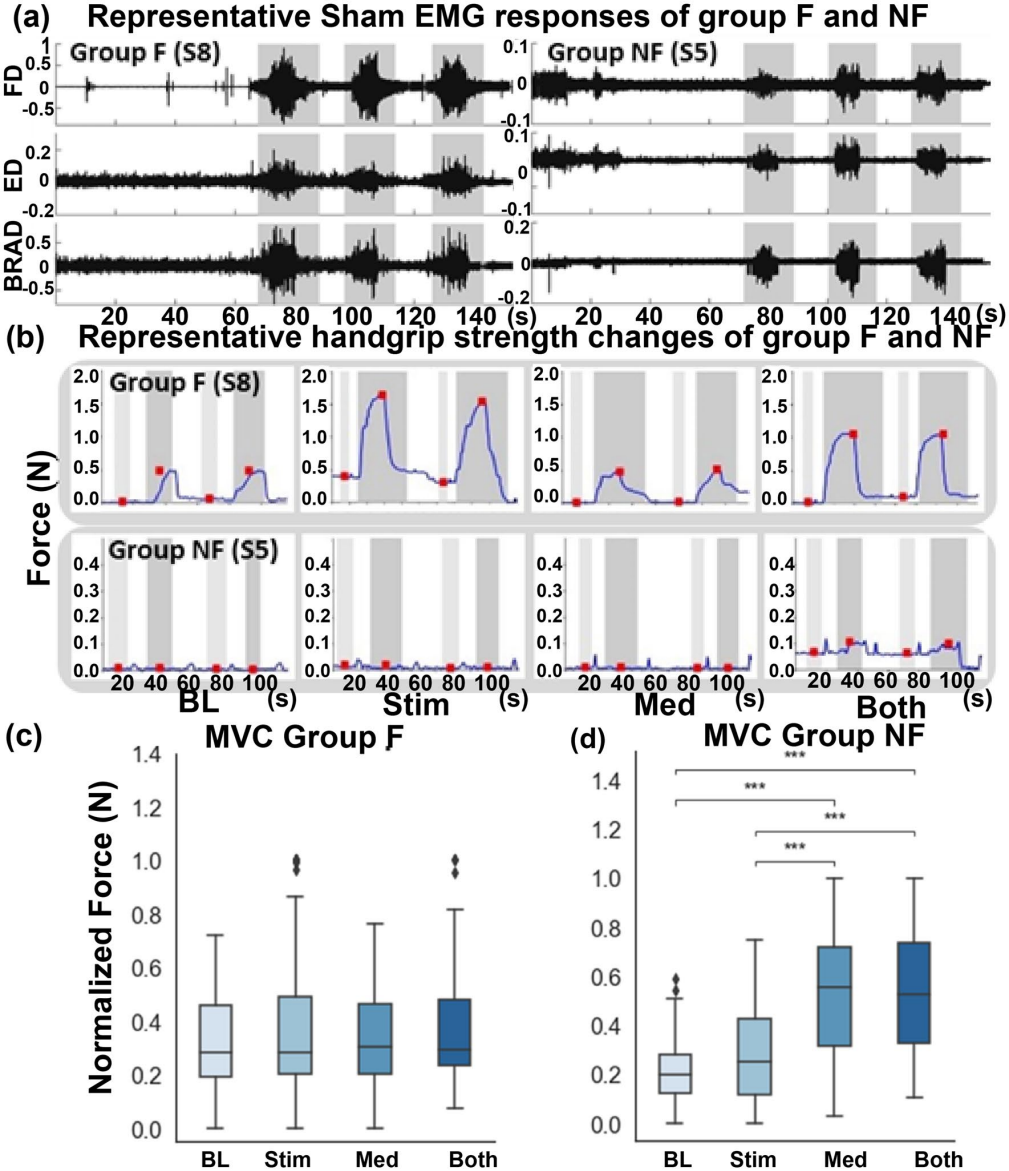
The performance of MVC test in Group F and NF. (**a**) Representative forelimb muscle EMG responses of one subject of each group (Group F: S8 and Group NF S5) during initial baseline testing. (**b**) Representative MVC results of the same subjects in the panel (**a**). Light gray bars indicate baseline measurement and dark gray bars indicate the time during the attempted pull. Red squares mark the baseline and the maximum values recorded (MVC: F, NF, n = 5 for each group). (**c**, **d**) Normalized MVC results from Group NF and Group F, respectively. The number of asterisks indicates the threshold significance level detected by three-way ANOVA; *: *P* < 0.05, **: *P* < 0.01, and ***: *P* < 0.001. Black diamonds indicate outlier values. Baseline (BL), stimulation only (Stim), medication only (Med) and combined stimulation and medication (Both). The means and the standard deviation (SD) are shown.

**Figure 4:**
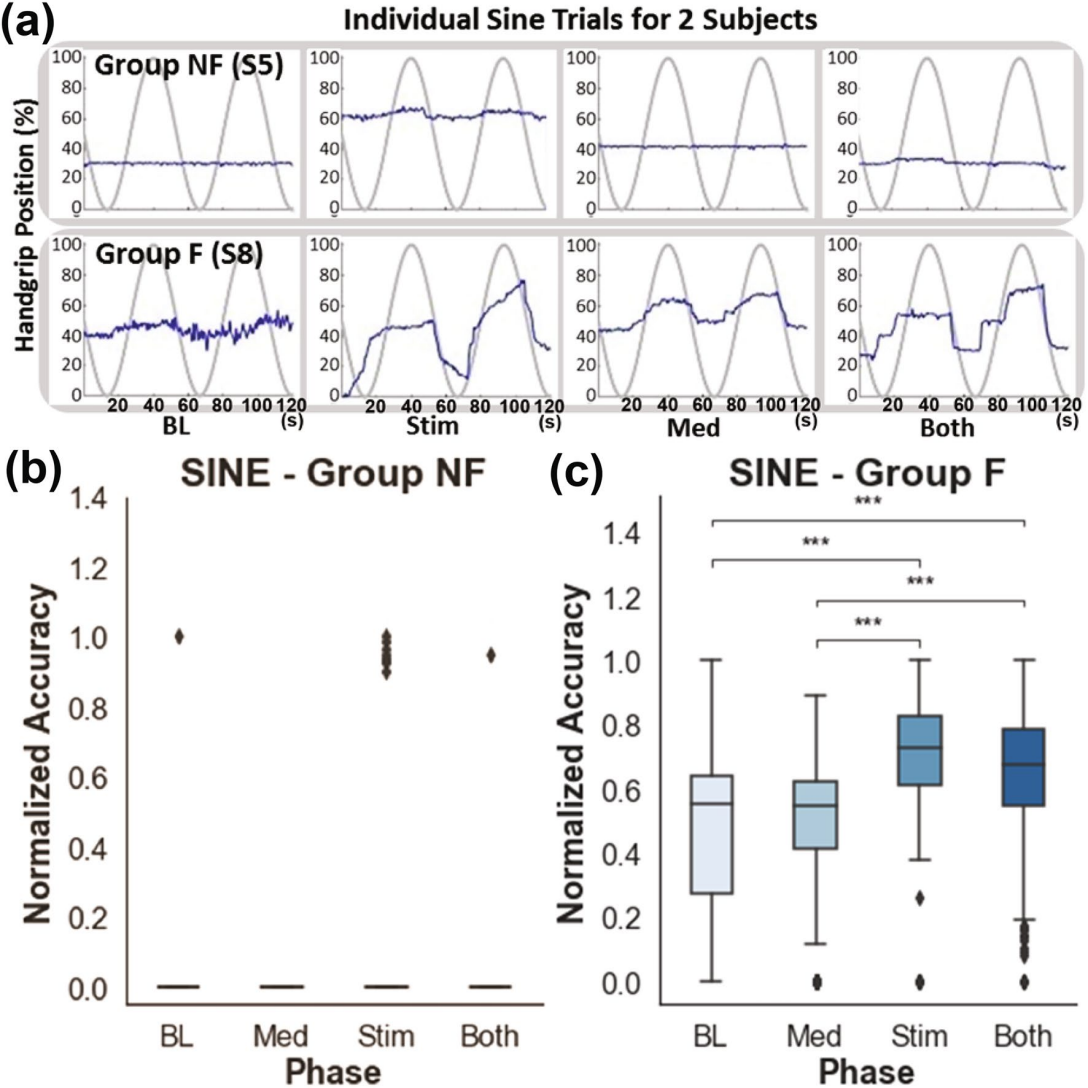
The performance of the SINE test in Group F and NF. (**a**) Representative results of SINE testing from two subjects, one from each group (Group F: S8 and Group NF S5). (**b**, **c**) Normalized sine scores for Group NF and Group F, respectively (n = 5 for each group). The number of asterisks indicates the threshold significance level detected by the three-way ANOVA; *: *P* < 0.05, **: *P* < 0.01, and ***: *P* < 0.001. The means and the standard deviation (SD) are shown.

**Figure 5:**
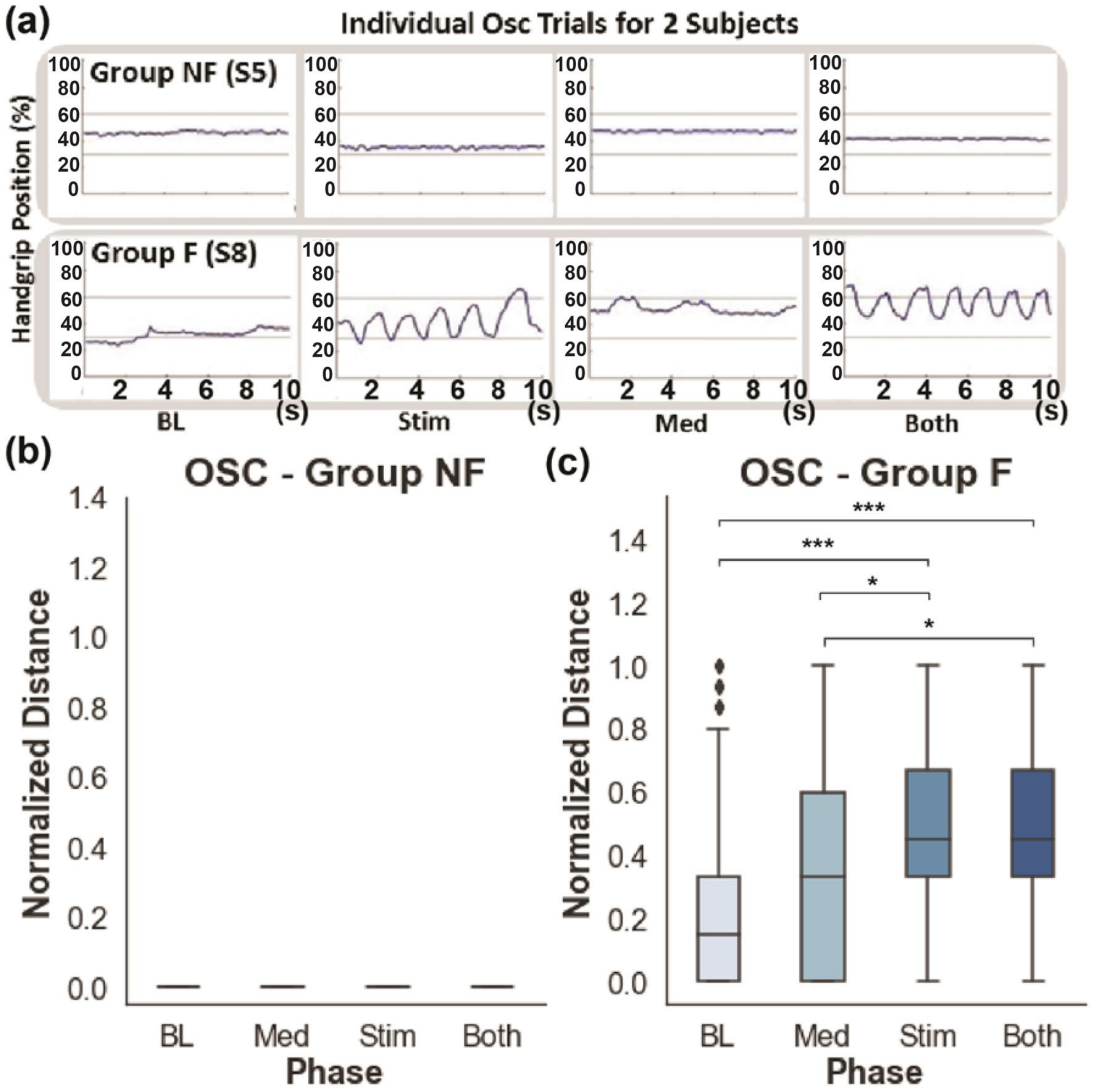
The performance of the OSC test in Group F and NF. (**a**) Representative results of OSC testing from two subjects, one from each group (Group F: S8 and Group NF S5). (**b**, **c**) Normalized oscillation scores for Group NF and Group F, respectively (n = 5 for each group). The number of asterisks indicates the threshold significance level detected by the two-way ANOVA; *: *P* < 0.05, **: *P* < 0.01, and ***: *P* < 0.001. The means and the standard deviation (SD) are shown.

The responses to tSCS, buspirone, or combined treatment varied as a function of the motor force group. In Group F, buspirone plus sham tSCS did not increase MVC nor improve the sine test compared to placebo and sham tSCS. However, buspirone plus sham tSCS was associated with improved OSC performance compared to the double placebo condition (*P* < 0.01). The combination of tSCS plus placebo drug significantly enhanced MVC force (Fig. 3; *P* = 0.001), the sine test (Fig. 4, *P* = 0.001) and the OSC rate (Fig. 5; *P* = 0.001) compared to sham treatment plus sham tSCS. Combining buspirone to tSCS did not further increase the MCV, the sine test force or the OSC rate beyond the effect of tSCS alone (Figs. 3, 4 and 5, respectively). There was no improvement in the NF group in any of the three motor tasks after any of the treatment conditions compared to sham treatment despite ongoing physical therapy. The level of injury of the subjects was between C3 and C7, where as the stimulation delivered was at the C4-5 level. The level of injury did not seem to modify the responses induced by tSCS. The primary determining factor of responsiveness to tSCS and training was the magnitude of the residual baseline hand grip force generated by the patient, which was greater than 0.1 N in those subjects who responded to the experimental interventions. These observations confirmed that tSCS and motor training improved hand function in subjects with some minimal residual grip force, but not in those without residual grip force.

### Grip strength at follow-up

**Figure 6:**
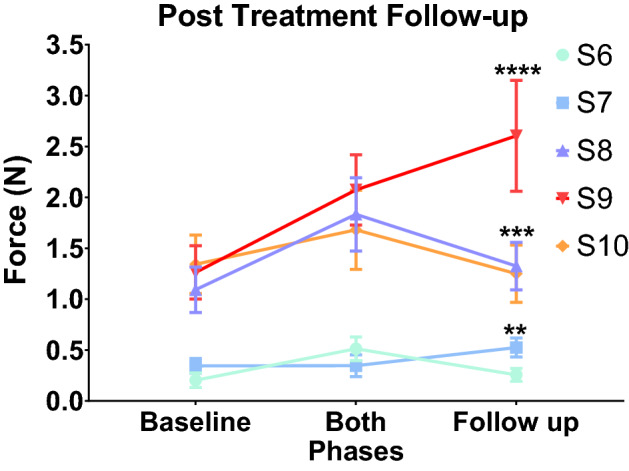
Follow-up MVC values when there was no stimulation or buspirone given to the patients in Group F 5 months after the last session of treatment. The MVC values of pre-treatment baseline (Baseline), the Both-phase (patients received both the stimulation and buspirone, Stim + Busp), and the follow-up washout sessions (Follow-up No Stim or Busp) were recorded from at least three different visits during each phase for subjects in Group F (**: *P* < 0.01 S7, ***: *P* < 0.05 S8, and ****: *P* < 0.0001 S9 when compared to the Baseline, detected by two-way ANOVA and Tukey’s test). The means and the standard deviation (SD) are shown.

To analyze the durability of post-treatment MVC scores after the tSCS intervention, we examined grip strength in subjects 2–5 months after the tSCS and buspirone treatments. These subjects received standard home physical therapy during this follow-up period, but they received no formalized, in-laboratory physical therapy. We observed no improvement in grip strength among subjects in Group NF, and the two subjects in Group F with the lowest hand grip forces reverted to baseline performance. However, three subjects (S7, S8, and S9) in Group F, who were at the higher end of grip force generation at the conclusion of the tSCS study (Fig. 6), maintained improved grip strength compared to their baseline performance despite the absence of ongoing tSCS (S7: *P* = 0.0056, S8: *P* = 0.0008, S9: *P* < 0.0001).

### Buspirone has more effect in modulating sensory perception and spasticity

**Figure 7:**
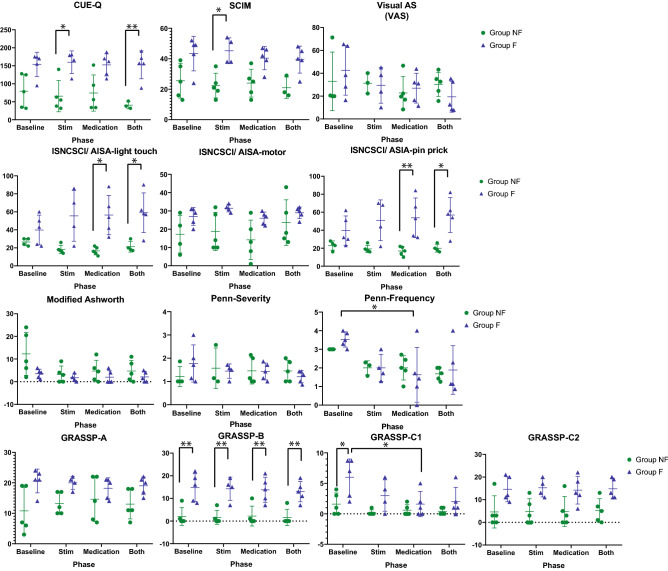
Results of the functional assessments. Standardized functional measures recorded at the end of each phase including baseline (BL), stimulation only (Stim), medication only (Med), and combined stimulation and medication (Both) for major functional test including AIS measurement for motor and sensory function, the Modified Ashworth scale of muscle tone, the self-assessed Penn spasm scale for frequency and severity of spasticity, and the self-assessed VAS as measure of spasticity. Treatment order is listed in the same order for each subject and does not reflect the actual order of treatment that each subject received. The number of asterisks indicates the threshold significance level detected by the two-way ANOVA; *: *P* < 0.05, **: *P* < 0.01, and ***: *P* < 0.001. The means and the standard deviation (SD) are shown.

While there were no observable trends in the responses of secondary outcomes to specific treatments, there was generally an improvement in functional assessments compared to baseline that was group dependent and present in Group F, but not in Group NF (Fig. 7). Two-way ANOVA and the post-hoc Tukey multiple comparison testing detected a statistically significant effect of treatment with buspirone only (within-subject factor) for the Penn-frequency (Group F, baseline vs Busp, *P* = 0.027), suggesting a decrease in the frequency of spasm.

The two-way ANOVA and the post-hoc Tukey multiple comparison testing also revealed study phase-by-group interactions for CUE-Q (Stim phase, NF vs F, *P* = 0.0215; Both phase, NF vs F, *P* = 0.0096), SCIM (Stim phase, NF vs F, *P* = 0.019), ISNCSCI /ASIA light touch (Medication phase, NF vs F, *P* = 0.016; Both phase, NF vs F, *P* = 0.0455), and ISNCSCI /ASIA pin pick (Medication phase, NF vs F: *P* = 0.0092; Both phase, NF vs F: *P* = 0.032). Notably, there was no significant difference between the NF and F groups during the baseline phase in any of the assessments above. Both groups also had no significant difference during all three treatment phases when compared to the baseline. These observations suggest that tSCS and/or buspirone induced opposite results of the above assessments in the NF and F groups.

The significant differences between the NF and F groups were detected in all phases of GRASSP sensation test (GRASSP-B) including the Baseline phase (Baseline phase, NF vs F: *P* = 0.0028; Stim phase, NF vs F: *P* = 0.0061; Medication phase, NF vs F: *P* = 0.0082; Both phase, NF vs F: *P* = 0.0098). The sensation of the fingertips evaluated by GRASSP B was consistently stronger in the F group than the NF group, regardless of the treatments. The GRASSP prehension ability (GRASSP-C1) of the F group was significantly higher than the NF group during the baseline phase (Baseline phase, NF vs F, *P* = 0.027), indicating better proprioceptive sensorimotor integration in the patients in the F group. However, the buspirone-only treatment significantly decreased the GRASSP-C1 scores of the F group compared to the baseline (F group, baseline vs. Medication: *P* = 0.026). Stimulation-only (Stim) and the combination of buspirone and stimulation (Both) also tended to decrease in the GRASSP-C1 scores of the F group. The GRASSP-C1 scores of the F group were no longer significantly higher than those of the NF group during Stim and Both phases.

## Discussion

Neuromodulation with electrical stimulation is a powerful intervention to facilitate rehabilitation of patients with SCI. Though various neuromodulatory methods have been used for decades, the optimal use of electrical stimulation is still evolving. Epidural stimulation of the cervical cord in the setting of SCI increased grip strength and fine control of hand movement. Non-invasive transcutaneous stimulation (tSCS) of the cervical cord with or without physical training also facilitated recovery of upper limb functions^10,11,16,24,25^. Transcutaneous SCS and buspirone also improved hand functions in a randomized, placebo-controlled study of patients with severe SCI^10^.

In the current study, all the enrolled patients had motor and sensory findings consistent with either ISNCSCI/AIS Grade A or B (nine ISNCSCI/AIS A and one ISNCSCI/AIS B), but half the patients had some residual muscle strength after receiving physical therapy alone (MCV > 0.1 and < 1.4 N) and the other half had no residual muscle strength despite months of physical therapy (MCV < 0.1 N). Administration of buspirone alone did not consistently increase muscle strength or hand coordination in either the F or NF Group. Transcutaneous SCS improved hand grip strength and hand coordination/control in the F Group, but not in the NF Group. Adding buspirone to tSCS provided no additional benefit to the F Group over that benefit available from tSCS alone. The functional assessments of motor performance and spasticity tended to follow the tests of hand grip strength and coordination: the F Group showed improvements across a variety of functional assessments, though the changes were modest at best, and the NF Group showed no changes in functional assessments despite receiving months of physical therapy. Grip force assessments were performed twice a week throughout the entire physical therapy period. This allowed the patients to practice with the assessment protocol as well as to evaluate whether physical therapy could further improve grip performance. The patients were then grouped based on their grip force from the hand grip assessments in the last week of the physical therapy period (Phase I).

While tSCS improved hand strength and motor control in patients with some residual hand strength, the improvement was less substantial than the previously reported two-fold increase in maximal hand strength^10,24^. This could be explained by two factors. First, the subjects in the present study were more severely injured compared to the previous study. The average baseline hand strength for all 10 subjects after physical therapy was below 1.4 N, which was less than the average 5 N grip strength in the previous study. Thus, we believe that some minimal residual hand function is necessary for improvement after tSCS and ongoing physical therapy. Moreover, the extent of improvement after tSCS may be proportional to the residual hand strength and coordination present following physical therapy. Three of the subjects (S7, S8, and S9) with residual hand force (Group F) maintained the improvement in hand function for up to 5 months after treatment without additional training or tSCS treatment (Fig. 6), indicating that those subjects with greater residual hand grip strength were also the subjects who seemed to derive sustained benefit from the tSCS intervention. Therefore, if subjects being studied have more severe deficits and less residual function, neuromodulation is less likely to be effective as the degree of residual motor function is indicative of the extent to which the neural structures controlling hand grip and coordination are preserved and available for neurorehabilitation. The mechanism of neuromodulatory rehabilitation relies on enhancing residual neural function and recruiting additional neural elements to support the accomplishment of motor tasks. For these mechanisms to operate, there must be some minimum residual neural elements to work with after the injury. We wondered if the severity of initial injury is predictive of the capacity for neurorehabilitative effect from tSCS, but magnetic resonance imaging (MRI) obtained early following acute spinal trauma did not strongly correlate with the chronic motor and sensory effects of SCI. Therefore, a grip force larger than 0.05–0.1 N may be a better indicator of patients’ capacity to gain motor function from tSCS following SCI.

We also investigated whether buspirone could facilitate functional benefits without or with stimulation in these patients with only minimal hand grip forces. Buspirone has been used previously in combination with SCS and training, but none of those studies investigated the relationship between the treatment effect and residual motor function. In the present evaluator blinded, cross-over study, buspirone was largely ineffective in modulating hand grip. However, buspirone significantly reduced the occurrence of spasms, especially in the F group (Fig. 7, Penn-frequency test, Baseline vs Medication), within which the subjects had less severe motor paralysis. Although the results from the Modified Ashworth and Penn-severity tests showed no significant decreases in the severity of the spasms in the NF and F groups, tSCS and buspirone tended to decrease the overall spasticity (Modified Ashworth and VAS) and the occurrence of spasms (Penn-frequency) in the NF group. Given that spasms significantly reduce quality of life in patients with SCI, the reduced spasms may represent a real benefit of buspirone and/or tSCS, even though that was not the main focus of this study.

There was a trend in Group F for a greater response to buspirone-only and stimulation plus buspirone in ISNCSCI/ASIA-light touch and ISNCSCI /ASIA-pin prick tests, which may indicate that buspirone improves sensory feedback in patients with limited residue hand motor function.

The limitations of this study are three-fold. First, the evaluators were blinded to treatment conditions and the order of the treatment, and subjects were blinded to the order of drug treatment. We tried to keep the subjects blind to the stimulation conditions by not informing subjects about the expected outcomes of the study and the onset or the intensity of the stimulation. However, as the stimulation intensity of sham versus treatment was detectably different, the subjects were not fully blinded to the treatment condition. Thus, the lack of sensory input from the sham session might be correlated with a low motivation level of the patients, which could lead to a relatively poor motor outcome. Second, this study offers a partial answer to the question whether buspirone may improve motor function in the setting of training and stimulation after SCI. Buspirone appeared to reduce spasms in the subjects in this study, but it did not improve grip function in these same subjects. Therefore, we may conclude that the dose of buspirone that we tested did not seem to ameliorate hand grip function either alone or in combination with tSCS, but a more effective buspirone dose may be found in subsequent studies. Last, although tSCS induced significant improvements in both the force and dexterity in hand grip, there was no statistically significant increase observed in the ISNCSCI /ASIA-motor and all four GRASSP measurements among different treatment groups. More research is required to further translate these tSCS-induced improvements to general hand functions for these subjects with severe SCI who have residual grip strength greater than 0.1 N.

### Summary

We demonstrated that a sham-controlled trial that tSCS combined with training can improve hand function, both strength and movement control, in severely impaired subjects with some detectable grip strength. These findings are consistent with two previous reports of improved motor function in response to tSCS combined with physical training. Unlike the preceding investigations, this study included subjects who had no baseline hand function. While the transcutaneous stimulation showed more significant effects improving motor function and the force of hand grip in the patients with SCI and some residual hand strength, buspirone had a bigger role in improving the sensory function of the hand. The combination of tSCS and buspirone did not show a significant additive effect compared to the tSCS only group. Most importantly, our study demonstrated that the extent of residual hand grip function can effectively predict the extent of rehabilitation in response to neuromodulation and medication in the chronic stage of SCI. This suggests a novel method of predicting the treatment responses and selecting therapies for patient with SCI years after the original injury.

## Data Availability

The datasets generated and analyzed during the current study are available from the corresponding author on reasonable request.
